# Prevalence of Overweight and Obesity in Pediatric Congenital Heart Disease: Associations with Hypertension and Echocardiographic Findings

**DOI:** 10.3390/children13060826

**Published:** 2026-06-18

**Authors:** Benedetta Leonardi, Federica Calì, Chiara Pierri, Ugo Giordano, Giovanni Di Salvo, Giovanni Antonelli, Giulio Calcagni, Marcello Chinali

**Affiliations:** 1 Bambino Gesù Children’s Hospital, IRCCS, 00165 Rome, Italy; federica1.cali@opbg.net (F.C.); chiara.pierri@opbg.net (C.P.); ugo.giordano@opbg.net (U.G.); giovanni.antonelli@opbg.net (G.A.); giulio.calcagni@opbg.net (G.C.); marcello.chinali@opbg.net (M.C.); 2 Paediatric Cardiology and Congenital Heart Disease, University of Padua and Pediatric Research Institute (IRP), Città Della Speranza, 35127 Padua, Italy; giodisal@yahoo.it

**Keywords:** congenital heart disease, pediatric obesity, arterial hypertension, left ventricular remodelling, echocardiography

## Abstract

**Highlights:**

**What are the main findings?**

- Excess body weight is common in pediatric CHD and is associated with a dose-dependent increase in systolic blood pressure (*p* < 0.001).
- Overweight and obesity are linked to early cardiac remodeling, including increased left ventricular mass (*p* = 0.007) and wall thickness (*p* < 0.001), despite preserved systolic function.
- The association between BMI, blood pressure, and remodeling is prominent in biventricular circulation but not in univentricular physiology.
- BMI-related effects vary across CHD subtypes, with consistent associations in TGA, coarctation, and RVOT abnormalities.

**What are the implications of the main findings?**

- Excess weight identifies a preclinical phenotype characterized by structural cardiac changes preceding functional impairment.
- Routine assessment and early management of BMI should be integrated into CHD follow-up to prevent adverse remodeling.
- Risk stratification in CHD should incorporate metabolic factors alongside anatomical and hemodynamic variables.
- Tailored prevention strategies may be particularly relevant in CHD subtypes exposed to increased afterload.

**Abstract:**

Background: The impact of overweight and obesity on blood pressure and cardiac remodelling in pediatric congenital heart disease (CHD) remains incompletely defined, particularly across different ventricular physiologies and cardiac anatomies. Aim: To assess the association of overweight and obesity with arterial blood pressure and cardiac remodelling in pediatric and young adult patients with CHD, according to ventricular physiology and cardiac anatomy. Methods: In this observational study, pediatric CHD patients undergoing clinical and echocardiographic evaluation were classified by weight status and ventricular physiology, with additional stratification by cardiac anatomy. Associations between body mass index (BMI), arterial hypertension, and echocardiographic parameters were analyzed. Results: A total of 451 patients were included (mean age 13.1 ± 3.9 years; 74 univentricular, 377 biventricular). Overall, 16% were overweight and 7% obese. Hypertension was present in 16% and increased across BMI categories (14%, 26%, and 50%). BMI was associated with blood pressure category (*p* < 0.001), higher systolic blood pressure (*p* < 0.001), and increased left ventricular (LV) mass (*p* = 0.007), interventricular septal thickness (*p* < 0.001), and posterior wall thickness (*p* < 0.001), without evidence of systolic dysfunction. In adjusted models, overweight/obesity remained associated with worse blood pressure classification, both as a three-category outcome (OR 2.1, 95% CI 1.4–3.2; *p* < 0.001) and as a binary outcome (OR 2.3, 95% CI 1.5–3.7; *p* < 0.001), as well as with higher systolic blood pressure (β = 5.1 mmHg, 95% CI 2.4–7.8; *p* < 0.001), left ventricular mass index (LVMI) (β = 10.0, 95% CI 4.3–15.8; *p* < 0.001), interventricular septal thickness at end-diastole (IVSd) (*p* < 0.001), and left ventricular posterior wall in diastole (LVPWd) (*p* < 0.001), but not with diastolic blood pressure or systolic function. No significant associations were observed in univentricular patients, whereas in biventricular circulation higher BMI was consistently associated with worse blood pressure and remodeling, without systolic dysfunction. Conclusions: Excess body weight is independently associated with adverse blood pressure status and early LV structural remodelling in pediatric and young adult patients with CHD, despite preserved systolic function. These findings support early cardiovascular risk surveillance and preventive strategies targeting overweight and obesity in CHD care.

## 1. Introduction

Over the past decades, advances in diagnostic, surgical, and follow-up strategies have markedly improved survival among children with congenital heart disease (CHD), shifting clinical attention toward long-term comorbidities and acquired cardiovascular risk factors. Among these, overweight and obesity have emerged as growing concerns, even in pediatric CHD populations [1,2,3,4]. Although nutritional management in children with CHD has historically focused on undernutrition and failure to thrive, contemporary data indicate that the prevalence of overweight and obesity in this population is broadly comparable to that observed in the general pediatric population [5,6,7], with substantial heterogeneity according to age, ethnicity, and disease severity.

Epidemiological evidence underscores the magnitude of this problem. In a systematic review including more than 21,000 patients, Willinger et al. [8] reported that, among children with CHD, the prevalence of overweight ranged from 9.5% to 31.5% and that of obesity from 9.5% to 26%. Similar findings were reported by Shustak et al. [9] and Steele et al. [3], who confirmed that overweight and obesity are common in children and adolescents with CHD. In adults with congenital heart disease, overweight prevalence ranged from 22% to 53%, while obesity prevalence ranged from 7% to 26%, suggesting that excess weight increases with age and remains a relevant issue throughout life. Recent data from the prospective multicenter PATHFINDER-CHD Registry further highlight the clinical relevance of excess body weight in adults with congenital heart disease, showing that nearly half of patients were overweight or obese and that higher BMI was significantly associated with arterial hypertension, diabetes mellitus, sleep apnoea, and greater use of heart failure–specific medications [10]. In addition, the burden of excess weight may vary across populations. In an Asian citywide school-based cohort, Chen et al. [11] showed that children with CHD were initially more frequently underweight and less frequently overweight or obese than healthy controls, particularly in the presence of moderate-to-severe or cyanotic CHD; however, during adolescence, the prevalence of overweight and obesity increased and became comparable to that observed in the general population. These findings suggest that nutritional trajectories in CHD may change over time, shifting from early growth impairment to excess weight later in life, as reiterated by Andonian et al. [12].

In addition to unhealthy dietary habits, parental overprotection may unintentionally limit daily physical activity in children with CHD, thereby promoting weight gain and adverse cardiovascular consequences over time. Barbiero et al. [13], in a cohort of 316 children and adolescents with CHD, reported an obesity prevalence of 26.9% and found that obesity was significantly associated with abnormal total cholesterol and triglyceride levels, as well as higher blood glucose and C-reactive protein levels. These findings suggest that patients with excess weight may also have a more unfavourable metabolic and inflammatory profile. Moreover, these patients more frequently had a family history of obesity, dyslipidaemia, diabetes, hypertension, and ischaemic heart disease.

Longitudinal data further reinforce this concern. Tamayo et al. [14] provided evidence that children with CHD experience a progressive increase in weight and BMI z-scores over time, indicating a significant risk of developing overweight and obesity during follow-up. In addition, excess weight was associated with lower exercise capacity and a higher systolic blood pressure response during exercise testing [14]. Similarly, Perin et al. [4] documented that children with excess weight had higher mean systolic blood pressure percentiles than normal-weight children, both among patients with CHD and among healthy controls, with the highest values observed in obese children compared with overweight children. In addition, Opp et al. [15] showed that adults with Fontan circulation and obesity have worse aerobic capacity and a more unfavourable haemodynamic profile, both at rest and during exercise, compared with patients with normal weight or overweight. These abnormalities resemble the obesity-related phenotype of heart failure with preserved ejection fraction, suggesting that obesity may represent a clinically relevant and potentially modifiable risk factor in Fontan patients. In repaired Tetralogy of Fallot, CMR studies have suggested an adverse impact of obesity on ventricular remodeling and function. Fogel et al. [16] reported increased biventricular mass, particularly involving the right ventricle, despite preserved ejection fraction and cardiac index, whereas Maskatia et al. [17] found lower biventricular systolic function and greater ventricular dilation in obese patients. In the study by Safwat Aly et al. [18], obesity was shown to adversely affect ventricular remodeling and functional capacity in pediatric patients with repaired Tetralogy of Fallot. Specifically, obesity was associated with larger biventricular volumes, lower right and left ventricular ejection fractions, and reduced exercise performance, even after adjustment for pulmonary regurgitation severity. In the study by Buelow et al. [19]., obesity adversely affected perioperative outcomes in adults with congenital heart disease undergoing pulmonary valve replacement, being associated with longer hospitalization and an increased risk of postoperative arrhythmias. Finally, in a prospective cohort of 1250 surgical cases, Radbill et al. showed that postoperative arrhythmias after congenital heart surgery increase progressively across BMI categories, reaching 45% in obese patients, and that obesity remains independently associated with arrhythmic risk after adjustment (OR 1.64, *p* = 0.036) [20]. These data suggest that excess body weight may contribute to postoperative electrical instability in children and young adults with CHD.

Despite this growing body of evidence, obesity remains under-recognized and under-managed in CHD care. Several authors [8,12,13,21] have emphasized the need for structured, multidisciplinary preventive strategies to identify and address excess weight from early life. Routine cardiology follow-up should therefore include regular assessment of weight status, blood pressure, lifestyle habits, and metabolic risk profile, together with targeted counselling on nutrition and physical activity. Nevertheless, data remain limited regarding the relationship between overweight/obesity, arterial hypertension, and echocardiographic markers of ventricular remodeling and dysfunction in pediatric CHD populations. Therefore, the present study aimed to evaluate the prevalence of overweight and obesity in a pediatric CHD cohort and to investigate their association with arterial hypertension and echocardiographic abnormalities, including ventricular mass and functional parameters.

## 2. Materials and Methods

### 2.1. Study Population and Phenotypic Classification of Congenital Heart Disease, Blood Pressure and Echocardiographic Assessment

Data from pediatric patients referred to our congenital heart disease day hospital were retrospectively collected. For each patient, body weight, height, and arterial blood pressure measurements obtained during a scheduled outpatient visit were analyzed. Blood pressure was measured in the right arm after 3–5 min of seated rest, using an appropriately sized cuff selected according to mid-upper arm circumference, with bladder width ≥ 40% and bladder length covering 80–100% of arm circumference. When elevated values were recorded, blood pressure was calculated as the average of three consecutive measurements. During the same visit, a comprehensive echocardiographic examination was performed according to institutional standard practice by an experienced team of sonographers and pediatric cardiologists with longstanding expertise in congenital heart disease imaging. The echocardiographic assessment included M-mode measurements of interventricular septal thickness in diastole (IVSd) and left ventricular posterior wall thickness in diastole (LVPWd). Left ventricular mass was evaluated both as an absolute value and after indexation to height raised to the power of 2.7 (LVMI, g/m^2.7^). Right ventricular systolic function was assessed using tricuspid annular plane systolic excursion (TAPSE) and right ventricular fractional area change (FAC), and, when available, three-dimensional RV function was also analyzed. Left ventricular systolic function and myocardial deformation were evaluated by three-dimensional echocardiography, including three-dimensional ejection fraction, global circumferential strain (LV 3D GCS), and global longitudinal strain (LV 3D GLS). When available, right ventricular free wall longitudinal strain (RVFWLS) was also assessed. The overall cohort was initially classified according to ventricular physiology into patients with single-ventricle physiology and those with biventricular circulation. Patients with biventricular circulation were subsequently further stratified into homogeneous subgroups based on the underlying cardiac anatomy. Specifically, patients were classified into the following categories: (i) transposition of the great arteries (TGA); (ii) right ventricular outflow tract (RVOT) abnormalities, including Tetralogy of Fallot (TOF) and related conditions such as pulmonary atresia with intact ventricular septum (PA-IVS), pulmonary valve stenosis (PVS), double-outlet right ventricle (DORV), and pulmonary atresia with ventricular septal defect (PA + VSD); and (iii) coarctation-related lesions (COA+), including coarctation of the aorta (COA), transposition of the great arteries associated with coarctation (TGA + COA), bicuspid aortic valve (BAV), aortic valve stenosis (AVS), and interrupted aortic arch (IAA). An additional group, called “other” included other congenital heart diseases not falling into the previously defined categories, such as anomalous pulmonary venous return (APVR), atrial septal defect (ASD), ventricular septal defect (VSD), Kawasaki disease, complete atrioventricular canal (CAV), coronary artery anomalies, mitral valve stenosis (MVS), truncus arteriosus, congenitally corrected transposition of the great arteries (ccTGA), Ebstein anomaly, and other congenital heart diseases not otherwise classified.

### 2.2. Statistical Methods

Weight status was classified according to the International Obesity Task Force (IOTF) reference cut-offs. For each patient, body mass index (BMI) was interpreted using age- and sex-specific IOTF thresholds, corresponding to the adult BMI values of 25 kg/m^2^ for overweight and 30 kg/m^2^ for obesity at 18 years of age. Based on these cut-offs, patients were classified as normal weight, overweight, or obese according to their BMI, age, and sex. This classification was applied to all paediatric and adolescent patients included in the study and was used for all analyses involving weight status. In additional analyses, overweight and obese patients were combined into a single overweight/obese group to assess the overall impact of excess body weight and to assess overall impact and improve statistical stability given the limited sample size of the obese subgroup and the biological continuum of adiposity-related risk.

Blood pressure categories were defined according to the 2017 American Academy of Pediatrics (AAP) Clinical Practice Guideline. In patients younger than 13 years, elevated blood pressure and hypertension were defined using age-, sex-, and height-specific percentiles, with elevated blood pressure corresponding to values ≥ 90th percentile and hypertension to values ≥ 95th percentile. For patients aged 13 years or older, fixed thresholds were used, with blood pressure ≥ 120/80 mmHg classified as elevated blood pressure and ≥ 130/80 mmHg classified as hypertension.

Categorical variables were analyzed using the chi-square test or Fisher’s exact test, as appropriate. When the chi-square test yielded significant results, standardized residuals were examined to identify the cells contributing to the association. Continuous variables were analyzed using non-parametric tests: the Wilcoxon rank-sum test for two-group comparisons and the Kruskal–Wallis test for comparisons across three groups. When the Kruskal–Wallis test was significant, post hoc pairwise comparisons were performed using Dunn’s test with Holm correction for multiple testing. Analyses were stratified by ventricular physiology, namely single-ventricle versus biventricular circulation, and by major CHD subgroups. Associations between weight status and echocardiographic parameters were evaluated using the same non-parametric approach.

To strengthen the analysis and account for potential confounders, multivariable regression models were performed. Linear regression models were used for continuous outcomes, including systolic and diastolic blood pressure values and echocardiographic parameters, and were adjusted for age, sex, and CHD diagnosis/severity. Results were reported as β coefficients with 95% confidence intervals. Categorical blood pressure outcomes were analyzed using ordinal logistic regression for the three-category blood pressure classification and binary logistic regression for the two-category hypertension/abnormal blood pressure classification; these models were adjusted for CHD diagnosis/severity. Age, sex, and height were not included as additional covariates because paediatric blood pressure categories already incorporate age-, sex-, and height-specific thresholds. Results were reported as odds ratios with 95% confidence intervals.

Additional multivariable linear regression analyses were performed to assess the relative contribution of BMI and systolic blood pressure to indexed left ventricular mass (LVMI). LVMI was used as the dependent variable, while BMI and systolic blood pressure were tested as predictors in models adjusted for age, sex, and CHD diagnosis/severity. A stepwise forward selection approach was applied to evaluate the incremental contribution of each variable through changes in R^2^. The association between systolic blood pressure and LVMI was also explored after stratification by BMI category, comparing normal-weight with overweight/obese patients.

Results were summarized in stratified tables using the gtsummary package and exported in Word format. A two-sided *p* value < 0.05 was considered statistically significant.

## 3. Results

A total of 451 pediatric patients (mean age 13.1 ± 3.9 years) with congenital heart disease were included in the study. The distribution of congenital heart disease subgroups differed significantly across weight categories (*p* < 0.05). Among normal-weight patients, RVOT abnormalities were the most frequent diagnosis (32%), followed by other CHD (30%) and COA+ (23%), while TGA accounted for 15% of cases. The distribution of anatomical diagnoses was similar in overweight and obese patients. “Other CHD” was the most frequent category in both groups (34% in both), followed by CoA+ (27% vs. 25%), RVOT abnormalities (25% vs. 22%), and TGA (14% vs. 19%). Overall, no specific CHD subgroup clearly predominated among patients with excess body weight.

### 3.1. Overall Population and Congenital Heart Disease Subgroup Analysis: Clinical and Echocardiographic Parameters Across Three BMI Categories (Normal Weight, Overweight, Obese)

**Figure 1:**
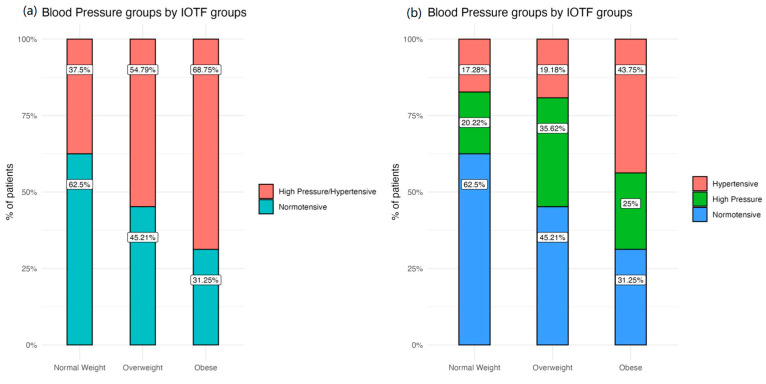
Distribution of blood pressure categories according to IOTF weight groups. Legend: (**a**) Stacked bar chart showing the percentage of normotensive versus high blood pressure/hypertensive patients across normal-weight, overweight, and obese groups. (**b**) Stacked bar chart showing the percentage of normotensive, high blood pressure, and hypertensive patients across the same IOTF weight categories. Percentages are expressed within each weight group. IOTF, International Obesity Task Force.

A total of 272 normal-weight, 73 overweight, and 32 obese patients were included in the analysis. The distribution of blood pressure categories significantly differed across the three groups (*p* < 0.001), with a progressive decrease in the proportion of normotensive subjects from 63% in the normal-weight group to 45% in the overweight group and 31% in the obese group, while the prevalence of hypertension increased, reaching 44% in obese patients compared to 17% in normal-weight individuals (Table 1). Similarly, when considering two categories, the prevalence of elevated blood pressure or hypertension increased from 38% in normal-weight individuals to 55% in overweight and 69% in obese patients (*p* < 0.001, specifically *p* < 0.001) (Figure 1).

**Figure 2:**
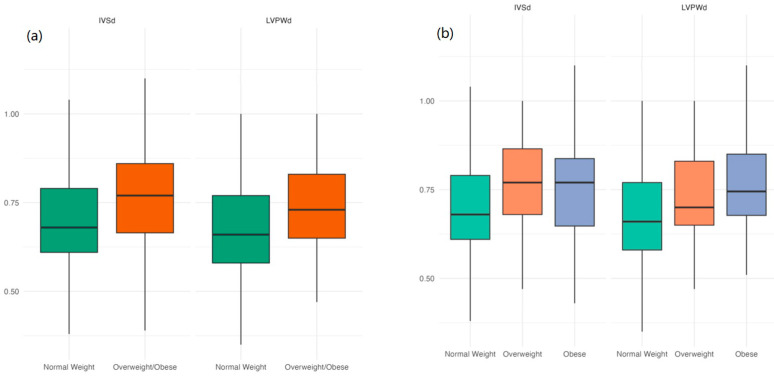
Distribution of left ventricular structural parameters according to IOTF weight groups. Legend: (**a**) Boxplots comparing normal-weight patients with the combined overweight/obese group for interventricular septal thickness in diastole (IVSd) and left ventricular posterior wall thickness in diastole (LVPWd). (**b**) Boxplots showing the distribution of the same parameters across the three IOTF categories: normal weight, overweight, and obese. In both panels, higher values of IVSd and LVPWd were observed in patients with excess body weight, supporting the presence of early concentric left ventricular remodeling. Boxplots show median, interquartile range, and range. IOTF, International Obesity Task Force.

Systolic blood pressure significantly increased across groups, with median values of 111 (102–120) mmHg in normal-weight, 119 (109–124) mmHg in overweight, and 121 (111–130) mmHg in obese subjects (*p* < 0.001), whereas no significant differences were observed for diastolic blood pressure (*p* = 0.399). Left ventricular mass index (LVMI) was significantly higher in overweight and obese patients compared to normal-weight individuals (*p* = 0.007). Similarly, IVSd and LVPWd showed a significant increase both when overweight and obese patients were combined into a single group and when the three weight categories—normal weight, overweight, and obese—were analyzed separately, with values increasing across groups (both *p* < 0.001) (Figure 2).

No significant differences were observed among groups in either left ventricular systolic function, assessed by LV 3D GLS, LVEF, and 3D LVEF, or right ventricular function, assessed by TAPSE, FAC, 3D RVEF, and RVFWLS (Table 1).

Patients were initially stratified according to ventricular physiology into univentricular and biventricular groups. The biventricular cohort was then subdivided according to the main anatomical diagnosis into four major CHD subgroups: CoA+, TGA, RVOT abnormalities, and other CHD diagnoses.

When BMI was analyzed in three categories within the biventricular anatomical subgroups, the relationship between BMI category and blood pressure profile varied across diagnoses (Table 1). The CoA+ subgroup included 62 normal-weight, 20 overweight, and 8 obese patients; the TGA subgroup included 40, 10, and 6 patients, respectively; the RVOT abnormality subgroup included 88, 18, and 7 patients; and the other CHD subgroup included 82, 25, and 11 patients. Overall, overweight and obese patients showed a lower proportion of normotensive subjects and a higher prevalence of elevated blood pressure and/or hypertension compared with normal-weight patients, although this pattern differed across anatomical subgroups. Using the three-category blood pressure classification, significant differences across BMI groups were observed in the CoA+ subgroup (*p* = 0.020), in the TGA subgroup (*p* = 0.014), and in patients with RVOT abnormalities (*p* = 0.017), but not in the other CHD subgroup. When elevated blood pressure and hypertension were combined into a single abnormal blood pressure category, the association remained significant only in patients with RVOT abnormalities (*p* = 0.011), suggesting that the relationship between BMI and blood pressure burden was most consistent in this subgroup.

Echocardiographic findings also differed across anatomical subgroups (Table 1). In CoA+ and TGA patients, BMI category was not associated with significant differences in LVMI, LV wall thickness, or ventricular functional parameters. In patients with RVOT abnormalities, IVSd differed significantly across BMI groups (*p* = 0.030), whereas LVMI and most ventricular functional parameters were comparable; however, 3D LVEF was slightly higher in overweight and obese patients compared with normal-weight patients (*p* = 0.031). In the other CHD subgroup, IVSd and LVPWd differed significantly across BMI categories (*p* = 0.010 and *p* = 0.012, respectively), whereas LVMI and ventricular functional parameters did not differ significantly. Overall, no consistent differences in left or right ventricular systolic function or strain parameters were observed across BMI categories within the anatomical subgroups.

### 3.2. Comparison Between Normal-Weight and Overweight/Obese Patients: Overall and Subgroup Analyses

**Figure 3:**
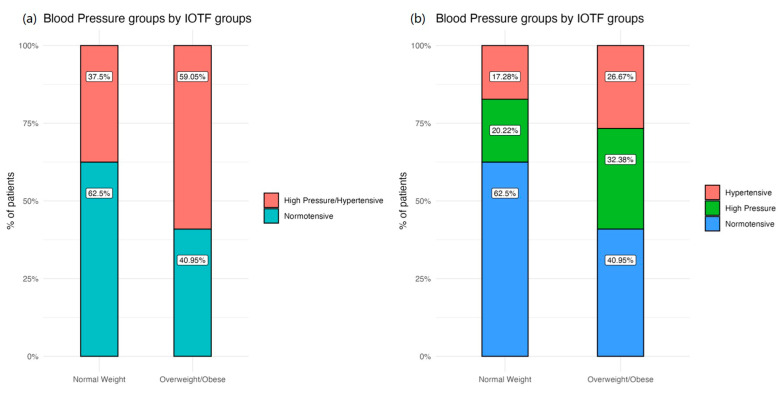
Distribution of blood pressure categories according to IOTF weight groups in normal-weight versus overweight/obese patients. Legend: (**a**) Stacked bar chart showing the percentage of normotensive patients and those with high blood pressure/hypertension in the normal-weight and overweight/obese groups. (**b**) Stacked bar chart showing the percentage of normotensive, high blood pressure, and hypertensive patients in the same two weight categories. Percentages are expressed within each weight group. IOTF, International Obesity Task Force.

To further explore the impact of excess body weight, overweight and obese patients were combined into a single group and compared with normal-weight individuals. In this analysis, 272 patients were of normal weight and 105 were overweight/obese. The distribution of blood pressure categories differed significantly between groups (*p* < 0.001), with a lower proportion of normotensive subjects in the overweight/obese group (41% vs. 63%) and a higher prevalence of both high blood pressure (32% vs. 20%) and hypertension (27% vs. 17%). This difference remained significant when blood pressure was analyzed using two categories (*p* < 0.001), with high blood pressure/hypertension observed in 59% of overweight/obese patients compared with 38% of normal-weight individuals (Figure 3). Systolic blood pressure was significantly higher in the overweight/obese group (*p* < 0.001), whereas diastolic blood pressure did not differ significantly (*p* = 0.747).

Structural cardiac parameters were also significantly increased in patients with excess weight, including LVMI (*p* = 0.002), IVSd (*p* < 0.001), and LVPWd (*p* < 0.001) (Figure 2). In contrast, no significant differences were observed in left or right ventricular systolic function or strain parameters (Table 2).

When overweight and obese patients were combined into a single excess-weight group and compared with normal-weight patients, subgroup analyses provided additional information and partly reinforced the findings observed in the three-category BMI analysis (Table 2). In the CoA+ subgroup, the difference in blood pressure distribution observed across the three BMI categories was no longer significant when using the three-category blood pressure classification. However, after dichotomizing blood pressure into normal versus abnormal values, overweight/obese patients showed a significantly higher prevalence of abnormal blood pressure compared with normal-weight patients (68% vs. 44%; *p* = 0.033). No significant differences in systolic or diastolic blood pressure values, LVMI, wall thickness, or ventricular functional parameters were observed.

In the TGA subgroup, the two-group analysis confirmed the significant difference in the three-category blood pressure distribution already observed in the three-group analysis (*p* = 0.011), mainly reflecting a lower proportion of normotensive subjects and a higher prevalence of elevated blood pressure among overweight/obese patients. However, this association was not maintained after dichotomization into normal versus abnormal blood pressure, and no significant differences were found in systolic or diastolic blood pressure values or in echocardiographic structural and functional parameters.

In patients with RVOT abnormalities, the two-group analysis confirmed and strengthened the findings of the three-category analysis. Overweight/obese patients showed a less favourable blood pressure profile, with significant differences both in the three-category classification (*p* = 0.042) and in the dichotomized analysis (56% vs. 31%; *p* = 0.020), together with higher systolic blood pressure (*p* = 0.024). Moreover, whereas the three-category analysis showed a significant difference only in IVSd, the two-group comparison demonstrated significantly higher LVMI, IVSd, and LVPWd in overweight/obese patients (*p* = 0.049, *p* = 0.026, and *p* = 0.030, respectively).

Finally, in the other CHD subgroup, the two-group analysis confirmed the absence of a significant association between excess body weight and blood pressure classification. However, consistent with the three-category analysis, overweight/obese patients showed greater LV wall thickness, with significantly higher IVSd and LVPWd (*p* = 0.003 and *p* = 0.013, respectively), whereas LVMI did not differ significantly. Across all anatomical subgroups, no consistent differences in left or right ventricular systolic function or strain parameters were observed between normal-weight and overweight/obese patients.

### 3.3. Multivariable Regression Analysis of Weight Status and Cardiovascular Outcome

In regression models adjusted for CHD diagnosis/severity, overweight/obesity was significantly associated with adverse blood pressure classification. In the model considering three blood pressure categories, overweight/obese patients had higher odds of being classified in a more unfavourable blood pressure category than normal-weight patients (OR = 2.1, 95% CI 1.4–3.2; *p* < 0.001). Similarly, when blood pressure status was analysed as a binary outcome, overweight/obesity was associated with higher odds of hypertension or abnormal blood pressure status (OR = 2.3, 95% CI 1.5–3.7; *p* < 0.001).

Consistently, in multivariable linear regression models adjusted for age, sex, and CHD diagnosis, overweight/obese patients showed significantly higher systolic blood pressure than normal-weight patients (β = 5.1 mmHg, 95% CI 2.4 to 7.8; *p* < 0.001), whereas no significant association was observed with diastolic blood pressure (β = 0.0 mmHg, 95% CI −2.0 to 2.1; *p* = 0.969). Overweight/obesity was also associated with higher left ventricular mass index (LVMI; β = 10.0, 95% CI 4.3 to 15.8; *p* < 0.001), greater interventricular septal thickness in diastole (IVSd; β = 0.1, 95% CI 0.0 to 0.1; *p* < 0.001), and greater left ventricular posterior wall thickness in diastole (LVPWd; β = 0.1, 95% CI 0.0 to 0.1; *p* < 0.001) (Table 3).

Conversely, no significant associations were observed between overweight/obesity and indices of ventricular systolic function or myocardial deformation, including LV 3D global longitudinal strain (β = 0.5, 95% CI −1.9 to 3.0; *p* = 0.658), conventional LVEF (β = −0.4, 95% CI −1.6 to 0.8; *p* = 0.555), TAPSE (β = −0.2, 95% CI −1.0 to 0.7; *p* = 0.705), fractional area change (β = 0.0, 95% CI −2.2 to 2.2; *p* = 0.996), 3D LVEF (β = −1.2, 95% CI −2.6 to 0.2; *p* = 0.099), 3D RVEF (β = −2.1, 95% CI −5.3 to 1.1; *p* = 0.203), or right ventricular free-wall longitudinal strain (β = 0.0, 95% CI −2.2 to 2.2; *p* = 0.996).

When weight categories were analysed separately, both overweight and obesity were associated with higher systolic blood pressure and greater left ventricular structural parameters compared with normal weight. Overweight patients had higher systolic blood pressure (β = 3.9 mmHg, 95% CI 0.8 to 7.0; *p* = 0.013) and higher LVMI (β = 8.9, 95% CI 2.3 to 15.4; *p* = 0.008), while obese patients showed higher systolic blood pressure (β = 7.8 mmHg, 95% CI 3.5 to 12.1; *p* < 0.001) and LVMI (β = 12.7, 95% CI 3.4 to 21.9; *p* = 0.008). IVSd and LVPWd were also significantly increased in both overweight and obese patients compared with normal-weight patients. Post hoc comparisons confirmed significant differences between normal-weight and overweight patients and between normal-weight and obese patients for systolic blood pressure, LVMI, IVSd, and LVPWd, whereas no significant differences were observed between overweight and obese patients (Table 4).

For categorical blood pressure outcomes, obesity was significantly associated with higher odds of belonging to a worse blood pressure category (OR = 3.8, 95% CI 1.9 to 7.8; *p* < 0.001), whereas the association for overweight was borderline (OR = 1.6, 95% CI 1.0 to 2.6; *p* = 0.051). When blood pressure status was analysed as a binary outcome, both overweight and obesity were associated with higher odds of hypertension or abnormal blood pressure status compared with normal weight (overweight: OR = 2.0, 95% CI 1.2 to 3.3; *p* = 0.012; obesity: OR = 3.6, 95% CI 1.7 to 8.2; *p* = 0.002).

In multivariable linear regression analysis adjusted for age, sex, and CHD diagnosis/severity, BMI was independently associated with higher indexed left ventricular mass (LVMI) (β = 1.2, 95% CI 0.8–1.5; *p* < 0.001), whereas systolic blood pressure was not significantly associated with LVMI in the fully adjusted model (β = 0.1, 95% CI −0.1 to 0.2; *p* = 0.307). Stepwise forward model selection further supported the predominant contribution of BMI to LVMI. The base model including age, sex, and CHD diagnosis/severity had an R^2^ of 0.126, which increased to 0.378 after adding BMI, compared with 0.151 after adding systolic blood pressure. When tested separately, both BMI and systolic blood pressure were significantly associated with LVMI. However, in subgroup analysis, systolic blood pressure was significantly associated with LVMI only in normal-weight patients (β = 0.2, 95% CI 0.0–0.4; *p* = 0.048), whereas no significant association was observed in overweight/obese patients (β = 0.0, 95% CI −0.4 to 0.3; *p* = 0.804).

In exploratory analyses restricted to patients with univentricular physiology, overweight/obesity was not significantly associated with either systolic or diastolic blood pressure after adjustment for age and sex. Compared with normal-weight patients, overweight/obese patients had similar systolic blood pressure (β = 0.1 mmHg, 95% CI −6.9 to 7.0; *p* = 0.988) and diastolic blood pressure (β = 1.3 mmHg, 95% CI −4.4 to 7.0; *p* = 0.639).

## 4. Discussion

### 4.1. Main Findings

This study confirms that overweight and obesity are becoming increasingly relevant and should not be underestimated in children and adolescents with congenital heart disease (CHD). In our cohort, approximately 28% of paediatric and adolescent patients with CHD were classified as overweight or obese, a prevalence broadly consistent with previous reported [3,8,9,13,14]. These data reinforce the concept that the global obesity epidemic also affects children with CHD and that excess body weight is no longer a marginal issue in this population.

More importantly, our study shows that overweight and obesity identify a subgroup of patients with CHD characterized by a less favourable cardiovascular profile. This profile was mainly defined by adverse blood pressure classification, higher systolic blood pressure, and early left ventricular structural remodelling. Therefore, excess body weight in children with CHD should not be considered only as a general paediatric comorbidity, but rather as an early and potentially modifiable cardiovascular risk factor.

### 4.2. Excess Body Weight and Blood Pressure Burden

In our cohort, excess body weight was associated with a higher burden of abnormal blood pressure, with a pattern that was more evident for systolic than for diastolic blood pressure. This finding is clinically relevant, as systolic blood pressure appears to be particularly sensitive to the haemodynamic effects of excess adiposity in pediatric CHD patients. Previous studies have reported similar observations [4,14]. Perin et al. [4] found higher systolic blood pressure percentiles in children with excess weight, both with and without CHD, while Tamayo et al. [14] showed that increasing BMI over time was associated with a higher systolic blood pressure response during exercise testing. In line with these studies, our results show that excess body weight is associated with a less favourable blood pressure profile in a heterogeneous pediatric CHD population, particularly through higher systolic blood pressure.

The graded pattern observed across BMI categories suggests that blood pressure burden increases along a continuum of adiposity. Obesity was associated with the strongest risk; however, overweight also showed an adverse profile, particularly when blood pressure was dichotomized as normal versus abnormal. This finding is noteworthy because overweight may be perceived as a less clinically relevant condition than obesity, whereas our results indicate that even moderate excess weight may be associated with early haemodynamic consequences in children and adolescents with CHD.

The association between excess body weight and abnormal blood pressure should also be interpreted within the broader context of obesity-related cardiometabolic risk. Barbiero et al. reported that, among children and adolescents with CHD, obesity was associated with a more unfavourable laboratory profile, including abnormal total cholesterol and triglyceride levels, higher blood glucose, and increased C-reactive protein [13]. These abnormalities may contribute to vascular dysfunction, low-grade inflammation, and increased blood pressure burden. In this perspective, the systolic blood pressure increase observed in our cohort may represent one clinical expression of a broader cardiometabolic phenotype.

The preferential association with systolic blood pressure may reflect the combined effects of increased circulating volume, higher cardiac output, sympathetic activation, vascular stiffness, endothelial dysfunction, and low-grade inflammation related to excess adiposity. In patients with CHD, these acquired mechanisms may add to pre-existing or residual loading abnormalities related to the underlying cardiac defect or previous surgical repair. Therefore, abnormal blood pressure in this population should not be interpreted only as an isolated acquired risk factor, but as a potentially relevant contributor to long-term cardiovascular burden.

### 4.3. Left Ventricular Remodelling and Preserved Systolic Function

An additional relevant observation of our study is the association between excess body weight and left ventricular structural remodeling in a large and heterogeneous pediatric cohort with congenital heart disease (CHD). Overweight and obese patients showed higher LVMI, greater interventricular septal thickness, and greater left ventricular posterior wall thickness compared with normal-weight patients. Similar findings were observed when overweight and obesity were analyzed as separate categories, with post-hoc comparisons confirming significant differences between normal-weight and overweight patients and between normal-weight and obese patients, but not between overweight and obese patients.

Importantly, our additional multivariable analyses suggest that BMI is the main determinant of LVMI in the overall cohort. After adjustment for age, sex, CHD diagnosis/severity, and systolic blood pressure, BMI remained significantly associated with LVMI, whereas systolic blood pressure did not show an independent effect in the fully adjusted model. Moreover, the inclusion of BMI substantially improved model fit compared with the inclusion of systolic blood pressure. These findings indicate that excess adiposity, rather than blood pressure alone, may play a predominant role in left ventricular mass increase in children and adolescents with CHD. The effect of systolic blood pressure appeared more limited and mainly evident among normal-weight patients. Taken together, these results suggest that even moderate excess body weight may be associated with measurable structural cardiac changes in this population, highlighting BMI as a key marker of adverse cardiac remodeling.

Our finding that excess body weight is associated with increased LVMI is consistent with previous literature showing a link between childhood adiposity and left ventricular remodeling [16,22,23]. Importantly, our multivariable analysis suggests that BMI may be a stronger determinant of LVMI than systolic blood pressure in this cohort. After adjustment for age, sex, CHD diagnosis/severity, and systolic blood pressure, BMI remained significantly associated with LVMI, whereas systolic blood pressure was not independently associated with LVMI in the fully adjusted model. This finding was further supported by the stepwise model selection, in which the inclusion of BMI substantially improved model fit compared with the inclusion of systolic blood pressure. These results suggest that excess adiposity, rather than blood pressure alone, may play a predominant role in the increase in left ventricular mass in children and adolescents with CHD. Although both obesity and blood pressure are known to contribute to cardiac remodeling, the relative predominance of BMI has not been extensively emphasized in children with CHD. Of note, the effect of systolic blood pressure appeared more limited and was mainly evident among normal-weight patients. Similar findings have been reported in pediatric populations with excess weight, in which BMI was identified as one of the strongest predictors of LVMI [24,25,26]. Our results extend this concept to a heterogeneous pediatric CHD cohort, suggesting that excess adiposity itself may play a central role in left ventricular remodeling, beyond the effect of blood pressure alone. Fogel et al. [16] demonstrated in patients with repaired Tetralogy of Fallot that obesity is associated with a significant increase in biventricular mass compared with normal-weight subjects, a pattern of hypertrophy that occurs independently of systolic hypertension. Similarly, in the pediatric population, Mannarino et al. [22] described a well-established relationship between body mass index and increased left ventricular size and mass. This association is biologically plausible, as excess adiposity increases metabolic demand, leading to expanded blood volume and greater cardiac preload; together with increased vascular afterload, these changes may promote myocardial remodeling. As also reported by Peterson et al. [23] in young adults, excess body weight can induce early concentric hypertrophic changes.

Interestingly, despite these structural abnormalities, our study did not identify any significant association between overweight/obesity and indices of ventricular systolic function or myocardial deformation, including conventional LVEF, 3D LVEF, 3D RVEF, TAPSE, FAC, LV 3D GLS, and right ventricular free-wall longitudinal strain.

This dissociation between left ventricular structural remodeling and preserved systolic function is supported by several cardiovascular imaging studies. Briston et al. [6] observed that, in adults with TOF, although obesity was associated with worse NYHA functional class, there were no statistically significant differences in left ventricular ejection fraction measured by echocardiography or cardiac magnetic resonance. Similarly, Simpson et al. [27] and Fogel et al. [16], evaluating CMR data in patients with repaired TOF, found no significant differences in LVEF or RVEF among normal-weight, overweight, and obese subjects.

However, the absence of abnormalities in conventional systolic indices or longitudinal strain should not be considered entirely reassuring. Rather, it may indicate an early and compensated phase of cardiovascular adaptation. In other words, excess body weight appears initially to be associated with increased systolic load and left ventricular remodeling before overt deterioration of biventricular systolic performance becomes detectable. The literature suggests that this structurally compensated phase may nevertheless conceal early physiological vulnerability.

First, myocardial impairment may involve strain components not captured by longitudinal deformation. Simpson et al. [27] showed that, despite preserved LVEF, obesity in patients with CHD was associated with significant abnormalities in global left ventricular circumferential strain, suggesting that myocardial dysfunction may begin through alterations in torsional and transverse shortening mechanics before longitudinal strain or ejection fraction decline becomes evident.

Second, functional reserve may be impaired. As demonstrated by Lewis et al. [28], an obesity-related remodelled myocardium may maintain a preserved ejection fraction at rest while showing blunted and inadequate systolic and diastolic responses during stress, thereby reducing the patient’s overall functional reserve.

Third, obesity may be associated with reduced myocardial metabolic efficiency. At the biochemical level, Peterson et al. [23] showed that, because of increased cardiac work, the obese heart may become insulin-resistant, require higher myocardial oxygen consumption, and become less efficient in converting chemical energy into mechanical work. These mechanisms may create the substrate for future pump dysfunction.

Overall, these data support the interpretation that the remodeling observed in our cohort represents an important warning signal. It may identify a therapeutic window during which lifestyle interventions could reverse or attenuate the hemodynamic burden of excess adiposity before the development of overt systolic dysfunction.

### 4.4. Biventricular and Univentricular Physiology

The separate evaluation of patients with biventricular and univentricular physiology provides an important additional perspective. In our cohort, overweight and obesity were less frequent among patients with univentricular physiology, and no statistically significant associations were observed between weight status and blood pressure outcomes in this subgroup. However, the directionally higher odds ratios for abnormal blood pressure in overweight and obese patients, together with the wide confidence intervals, suggest that these findings should be interpreted with caution rather than as evidence of no association. The lack of statistical significance may be partly explained by the small sample size, the low prevalence of excess body weight, and the marked clinical heterogeneity of univentricular patients, including differences in haemodynamic status, surgical stage, ventricular morphology, and medical treatment.

Differences between univentricular and biventricular patients may also reflect distinct clinical trajectories. Patients with univentricular physiology often have greater disease complexity, closer medical surveillance, persistent functional limitations, and more complex haemodynamic conditions, all of which may influence growth, body weight, and cardiometabolic risk. Conversely, patients with biventricular circulation may progressively adopt lifestyles more like those of their healthy peers, becoming more exposed to obesogenic behaviours. This may partly explain why the association between excess weight, blood pressure burden, and cardiac structural changes was more evident among patients with biventricular circulation. Within the biventricular cohort, the association between excess body weight and cardiovascular abnormalities was not uniform across anatomical diagnoses. The signal appeared more evident in patients with RVOT abnormalities, a subgroup that includes rTOF and related right ventricular outflow tract lesions. In these patients, overweight/obesity was associated with a higher prevalence of abnormal blood pressure, higher systolic blood pressure, and increased LV structural parameters, including LVMI, IVSd, and LVPWd. This finding is consistent with previous studies in this population showing that obesity is associated with increased biventricular mass, larger ventricular size, impaired ventricular function, and reduced exercise performance, suggesting that excess body weight may add a relevant haemodynamic and functional burden in this population [16,17,18]. Accordingly, patients with RVOT abnormalities may represent a subgroup in which careful cardiometabolic surveillance is particularly important.

### 4.5. Pathophysiological Interpretation and Relationship with Previous Studies

From a pathophysiological perspective, our findings are consistent with the known cardiovascular effects of obesity. Excess adiposity increases circulating blood volume, cardiac output, neurohormonal activation, systemic inflammation, and arterial load. In children with structurally normal hearts, these mechanisms contribute to hypertension and left ventricular hypertrophy. In patients with CHD, the same mechanisms may be particularly relevant because they act on a cardiovascular system that may already be affected by residual lesions, altered loading conditions, previous interventions, or reduced functional reserve. Therefore, obesity may act as an additional acquired stressor superimposed on congenital cardiovascular vulnerability.

The predominantly left-sided pattern of structural remodelling observed in our cohort is clinically meaningful. Increased LVMI and increased septal and posterior wall thickness suggest a pattern of early concentric adaptation, likely driven by higher systolic pressure and increased afterload. The absence of significant abnormalities in systolic function should not be interpreted as fully reassuring, but rather as an indication that these patients may still be in a preclinical and compensated stage. The relatively young age of our cohort may explain why functional deterioration was not yet evident, while structural markers were already detectable.

Our findings are in line with previous studies showing that obesity in children with CHD is associated with higher blood pressure and reduced exercise capacity [4,14,21]. Barbiero et al. [13] further highlighted that excess weight in children with CHD is often associated with clustering of additional cardiovascular risk factors, including dyslipidemia, impaired glucose metabolism, systemic inflammation, sedentary behaviour, passive smoking, and a positive family history of chronic non-communicable diseases. Our study extends this evidence by integrating blood pressure categories, continuous blood pressure values, and echocardiographic markers of ventricular remodelling within the same paediatric CHD cohort.

### 4.6. Clinical Implications and Future Perspectives

These findings have important clinical implications. Routine follow-up of children and adolescents with CHD should include systematic assessment of weight status and blood pressure using paediatric-appropriate definitions. In patients with excess body weight, particular attention should be paid to systolic blood pressure and early echocardiographic markers of left ventricular remodelling, even when systolic function is preserved. Early identification of this phenotype may allow timely lifestyle counselling and targeted preventive strategies before functional impairment develops.

Multidisciplinary care may be especially important in this setting. Paediatric cardiologists, nutritionists and exercise specialists should work together to promote healthy dietary habits, safe physical activity, and reduction of sedentary behaviors. This is particularly relevant because physical activity may be unnecessarily restricted in some children with CHD because of parental anxiety or uncertainty regarding exercise safety. Individualized exercise counselling, adapted to CHD phenotype and functional status, may help reduce long-term cardiometabolic risk.

Future longitudinal studies are needed to determine whether the structural changes observed in childhood persist or progress into adulthood, and whether they predict later ventricular dysfunction, exercise limitation, arrhythmias, or adverse cardiovascular outcomes. Further research should also clarify whether early weight management, blood pressure control, and structured physical activity interventions can reverse or attenuate left ventricular remodelling in paediatric CHD. Dedicated studies in patients with univentricular physiology are also warranted, given the limited sample size and exploratory nature of the present subgroup findings.

## 5. Limitations

This study should be interpreted in light of several limitations. First, its retrospective, single-center, observational design does not allow causal inference and may have introduced selection bias. Second, advanced echocardiographic parameters were not available in all patients. Third, despite the overall sample size, the obese subgroup and several lesion-specific subgroups remained relatively small, limiting statistical power and requiring cautious interpretation of subgroup findings. Fourth, the assessment of echocardiographic parameters was performed by an experienced team of sonographers with longstanding expertise in congenital heart disease. However, the studies were not all performed by the same operator, and formal inter-operator variability was not specifically assessed. Fifth, the cross-sectional design and the lack of longitudinal follow-up preclude conclusions regarding reversibility, progression over time, and long-term clinical impact; therefore, the observed structural abnormalities should be interpreted as a preclinical remodeling phenotype rather than definitive evidence of temporal disease progression. Finally, socioeconomic status was not evaluated, and a standardized questionnaire on daily physical activity was not administered to all patients.

## 6. Conclusions

Overall, our study shows that overweight and obesity in paediatric CHD are associated with adverse blood pressure classification, higher systolic blood pressure, and early left ventricular structural remodelling, without evidence of overt biventricular systolic dysfunction. These findings support the concept that excess body weight represents an early, measurable, and potentially modifiable cardiovascular risk factor in children and adolescents with CHD.

## Figures and Tables

**Table 1:** Clinical and instrumental characteristics of the study population stratified by body mass index categories (normal weight, overweight, and obese).

Variable	Normal Weight	Overweight	Obese	*p*-Value
**OVERALL**				
Age (years)	13.4 (10.1, 16.2)	13.7 (11.3, 15.7)	12.3 (9.6, 16.6)	0.935
Hypertension (3 groups)				**<0.001**
Normotensive	170 (63%)	33 (45%)	10 (31%)	
High Pressure	55 (20%)	26 (36%)	8 (25%)	
Hypertensive	47 (17%)	14 (19%)	14 (44%)	
Hypertension (2 groups)				**<0.001**
Normotensive	170 (63%)	33 (45%)	10 (31%)	
High Pressure/Hypertensive	102 (38%)	40 (55%)	22 (69%)	
Systolic Blood Pressure	111 (102, 120)	119 (109, 124)	121 (111, 130)	**<0.001**
Diastolic Blood Pressure	63 (60, 68)	62 (57, 70)	64 (60, 70)	0.399
LVMI (g/m^2^)	38 (30, 53)	52 (43, 65)	44 (41, 80)	**0.006**
IVSd (cm)	0.68 (0.61, 0.79)	0.77 (0.68, 0.87)	0.77 (0.65, 0.85)	**<0.001**
LVPWd (cm)	0.67 (0.58, 0.77)	0.71 (0.65, 0.83)	0.75 (0.68, 0.85)	**<0.001**
LV 3D GLS (%)	−22.9 (−25.1, −20.3)	−22.0 (−25.0, −20.2)	−22.4 (−25.4, −21.0)	0.689
LVEF (%)	62.0 (59.8, 65.2)	61.2 (59.9, 65.0)	62.8 (59.6, 66.0)	0.775
TAPSE (cm)	1.80 (1.50, 2.15)	1.80 (1.50, 2.40)	1.90 (1.60, 2.30)	0.484
FAC (%)	44.6 (41.0, 48.4)	45.3 (40.6, 48.4)	45.0 (41.9, 52.4)	0.897
3D LVEF (%)	62.5 (60.0, 65.0)	61.3 (59.4, 63.6)	62.5 (60.4, 64.4)	0.170
3D RVEF (%)	52.8 (48.1, 57.5)	50.4 (46.0, 58.7)	45.9 (45.5, 46.2)	0.199
RVFWLS (%)	−22.8 (−26.5, −20.6)	−24.1 (−26.1, −20.6)	−20.2 (−24.0, −16.3)	0.618
**COA+**				
Age (years)	13.6 (10.4, 16.3)	13.9 (12.1, 16.5)	14.5 (11.3, 17.6)	0.542
Hypertension (3 groups)				**0.020**
Normotensive	35 (56%)	7 (35%)	2 (25%)	
High Pressure	15 (24%)	10 (50%)	1 (13%)	
Hypertensive	12 (19%)	3 (15%)	5 (63%)	
Hypertension (2 groups)				0.121
Normotensive	35 (56%)	7 (35%)	2 (25%)	
High Pressure/Hypertensive	27 (44%)	13 (65%)	6 (75%)	
Systolic Blood Pressure	112 (101, 125)	120 (117, 125)	125 (111, 131)	0.253
Diastolic Blood Pressure	61 (57, 69)	60 (58, 70)	64 (60, 70)	0.805
LVMI (g/m^2^)	52 (34, 73)	58 (50, 64)	96 (96, 96)	0.289
IVSd (cm)	0.72 (0.61, 0.83)	0.78 (0.69, 0.90)	0.79 (0.65, 0.97)	0.215
LVPWd (cm)	0.68 (0.60, 0.82)	0.70 (0.66, 0.86)	0.75 (0.68, 0.93)	0.200
LV 3D GLS (%)	−22.3 (−25.6, −20.3)	−24.6 (−25.2, −19.3)	−25.4 (−27.4, −23.9)	0.500
LVEF (%)	63.7 (59.9, 67.2)	60.9 (59.6, 64.5)	60.2 (59.8, 64.9)	0.303
TAPSE (cm)	2.05 (1.65, 2.50)	2.10 (1.70, 2.35)	2.20 (2.00, 2.70)	0.751
FAC (%)	45.9 (42.3, 49.1)	41.7 (40.6, 48.2)	NA (NA, NA)	0.221
3D LVEF (%)	64.0 (60.9, 66.6)	60.9 (59.6, 63.6)	63.2 (60.4, 64.2)	0.154
3D RVEF (%)	52.5 (48.6, 56.6)	56.0 (49.3, 61.1)	NA (NA, NA)	0.386
RVFWLS (%)	−23.60 (−24.95, −21.60)	−24.50 (−26.40, −23.40)	NA (NA, NA)	0.386
**TGA**				
Age (years)	12.3 (10.3, 14.7)	15.0 (11.9, 17.1)	11.2 (7.8,12.0)	0.293
Hypertension (3 groups)				**0.014**
Normotensive	25 (63%)	3 (30%)	3 (50%)	
High Pressure	2 (5.0%)	5 (50%)	1 (17%)	
Hypertensive	13 (33%)	2 (20%)	2 (33%)	
Hypertension (2 groups)				0.198
Normotensive	25 (63%)	3 (30%)	3 (50%)	
High Pressure/Hypertensive	15 (38%)	7 (70%)	3 (50%)	
Systolic Blood Pressure	113 (106, 120)	125 (114, 126)	117 (110, 135)	0.166
Diastolic Blood Pressure	63 (60, 67)	60 (55, 66)	66 (60, 70)	0.252
LVMI (g/m^2^)	44 (38, 52)	65 (57, 72)	54 (34, 73)	0.278
IVSd (cm)	0.71 (0.61, 0.75)	0.71 (0.65, 0.81)	0.79 (0.59, 0.82)	0.712
LVPWd (cm)	0.66 (0.56, 0.77)	0.77 (0.65, 0.83)	0.79 (0.56, 0.85)	0.292
LV 3D GLS (%)	−22.75 (−24.80, −19.00)	−21.80 (−25.60, −20.30)	−21.20 (−22.40, 19.10)	0.769
LVEF (%)	61.6 (59.6, 64.4)	59.3 (58.6, 61.0)	64.9 (60.5, 65.5)	0.226
TAPSE (cm)	1.50 (1.30, 1.90)	1.80 (1.40, 2.40)	1.55 (0.98, 2.10)	0.754
FAC (%)	45 (41, 49)	47 (38, 50)	43 (40, 53)	0.943
3D LVEF (%)	61.60 (59.80, 62.90)	64.75 (58.25, 67.10)	60.70 (60.70, 61.90)	0.608
3D RVEF (%)	57.7 (50.6, 58.9)	46.3 (41.5, 53.9)	45.5 (45.5, 45.5)	0.172
RVFWLS (%)	−22.4 (−26.3, −21.4)	−20.5 (−22.9, −15.2)	−24.0 (−24.0, −24.0)	0.308
**RVOT ABNORMALITIES**				
Age (years)	13.7 (10.0, 16.5)	12.0 (9.1, 14.1)	12.3 (8.8, 17.5)	0.227
Hypertension (3 groups)				**0.017**
Normotensive	61 (69%)	10 (56%)	1 (14%)	
High Pressure	19 (22%)	4 (22%)	4 (57%)	
Hypertensive	8 (9.1%)	4 (22%)	2 (29%)	
Hypertension (2 groups)				**0.011**
Normotensive	61 (69%)	10 (56%)	1 (14%)	
High Pressure/Hypertensive	27 (31%)	8 (44%)	6 (86%)	
Systolic Blood Pressure	111 (103, 120)	117 (109, 123)	121 (112, 126)	0.053
Diastolic Blood Pressure	63 (60, 70)	63 (59, 71)	67 (59, 70)	0.830
LVMI (g/m^2^)	37 (24, 41)	47 (34, 53)	37 (29, 44)	0.234
IVSd (cm)	0.69 (0.60, 0.77)	0.82 (0.66, 0.92)	0.70 (0.59, 0.77)	**0.030**
LVPWd (cm)	0.67 (0.58, 0.77)	0.76 (0.68, 0.83)	0.73 (0.61, 0.76)	**0.098**
LV 3D GLS (%)	−22.8 (−24.5, −20.3)	−21.2 (−22.7, −19.2)	−29.4 (−29.4, −29.4)	0.109
LVEF (%)	62.0 (60.0, 64.1)	60.1 (59.9, 61.2)	64.9 (41.0, 70.1)	0.160
TAPSE (cm)	1.70 (1.40, 1.90)	1.65 (1.50, 1.75)	1.60 (1.40, 2.40)	0.996
FAC (%)	45.2 (41.1, 48.5)	46.0 (41.5, 48.4)	52.4 (52.4, 52.4)	0.339
3D LVEF (%)	62.4 (60.0, 64.3)	60.6 (59.5, 61.9)	66.5 (64.4, 68.6)	**0.031**
3D RVEF (%)	52.5 (48.1, 57.1)	49.7 (45.4, 57.9)	46.2 (46.2, 46.2)	0.420
RVFWLS (%)	−23.5 (−26.9, −20.8)	−24.5 (−26.8, −19.7)	−16.3 (−16.3, −16.3)	0.274
**OTHER**				
Age (years)	13.7 (10.4, 16.3)	13.7 (12.0, 15.7)	12.2 (9.8, 16.5)	0.989
Hypertension (3 groups)				0.313
Normotensive	49 (60%)	13 (52%)	4 (36%)	
High Pressure	19 (23%)	7 (28%)	2 (18%)	
Hypertensive	14 (17%)	5 (20%)	5 (45%)	
Hypertension (2 groups)				0.303
Normotensive	49 (60%)	13 (52%)	4 (36%)	
High Pressure/Hypertensive	33 (40%)	12 (48%)	7 (64%)	
Systolic Blood Pressure	110 (100, 120)	116 (109, 123)	120 (109, 130)	0.086
Diastolic Blood Pressure	64 (60, 67)	65 (55, 70)	61 (60, 70)	0.745
LVMI (g/m^2^)	37 (29, 52)	45 (43, 51)	44 (41, 80)	0.421
IVSd (cm)	0.66 (0.58, 0.72)	0.76 (0.68, 0.79)	0.80 (0.68, 0.92)	**0.010**
LVPWd (cm)	0.62 (0.55, 0.73)	0.69 (0.62, 0.73)	0.80 (0.67, 0.92)	**0.012**
LV 3D GLS (%)	−23.5 (−25.6, −21.2)	−22.2 (−24.1, −20.8)	−21.2 (−22.3, −19.1)	0.168
LVEF (%)	61.5 (59.1, 65.2)	64.8 (61.6, 66.3)	62.8 (58.5, 67.4)	0.149
TAPSE (cm)	1.80 (1.50, 2.20)	2.00 (1.50, 2.40)	1.90 (1.80, 2.30)	0.878
FAC (%)	41.0 (38.7, 46.6)	44.5 (39.4, 46.9)	45.0 (41.9, 47.0)	0.646
3D LVEF (%)	62.2 (59.9, 64.8)	61.7 (60.4, 63.6)	62.2 (58.3, 63.2)	0.671
3D RVEF (%)	52.7 (44.2, 58.6)	57.8 (57.8, 57.8)	NA (NA, NA)	0.513
RVFWLS (%)	−20.5 (−28.9, −20.2)	−24.4 (−31.2, −22.4)	NA (NA, NA)	0.210

^1^n (%); median (Q1, Q3); categorical variables were compared using Pearson’s chi-squared test, while continuous variables were analyzed using the Kruskal–Wallis test. Legend: LV 3D GLS, left ventricular three-dimensional global longitudinal strain; LVEF, left ventricular ejection fraction; 3D LVEF, three-dimensional left ventricular ejection fraction; TAPSE, tricuspid annular plane systolic excursion; FAC, fractional area change; 3D RVEF, three-dimensional right ventricular ejection fraction; RVFWLS, right ventricular free wall longitudinal strain; LVMI, left ventricular mass index; IVSd, interventricular septal thickness in diastole; LVPWd, left ventricular posterior wall thickness in diastole; NA, not available.

**Table 2:** Clinical and instrumental characteristics of the study population stratified by body mass index categories in normal weight and overweight/obese.

Variable	Normal Weight N = 272 ^1^	Overweight/Obese N = 105 ^1^	*p*-Value
**OVERALL**			
Age (years)	13.4 (10.1, 16.2)	13.3 (10.9, 15.8)	0.936
Hypertension (3 groups)			<**0.001**
Normotensive	170 (63%)	43 (41%)	
High Pressure	55 (20%)	34 (32%)	
Hypertensive	47 (17%)	28 (27%)	
Hypertension (2 groups)			<**0.001**
Normotensive	170 (63%)	43 (41%)	
High Pressure/Hypertensive	102 (38%)	62 (59%)	
Systolic Blood Pressure	111 (102, 120)	120 (110, 125)	<**0.001**
Diastolic Blood Pressure	63 (60, 68)	63 (59, 70)	0.747
LVMI (g/m^2^)	38 (30, 53)	50 (42, 66)	**0.002**
IVSd (cm)	0.68 (0.61, 0.79)	0.77 (0.65, 0.86)	**<0.001**
LVPWd (cm)	0.66 (0.58, 0.77)	0.73 (0.65, 0.83)	<**0.001**
LV 3D GLS (%)	−22.9 (−25.1, −20.3)	−22.0 (−25.0, −20.3)	0.442
LVEF (%)	62.0 (59.8, 65.2)	61.4 (59.9, 65.2)	0.707
TAPSE (cm)	1.80 (1.50, 2.15)	1.80 (1.55, 2.35)	0.229
FAC (%)	44.6 (41.0, 48.4)	45.3 (41.5, 48.4)	0.812
3D LVEF (%)	62.5 (60.0, 65.0)	61.6 (59.5, 63.7)	0.087
3D RVEF (%)	52.8 (48.1, 57.5)	49.7 (45.6, 57.8)	0.326
RVFWLS (%)	−22.8 (−26.5, −20.6)	−24.0 (−25.7, −20.6)	0.955
**TGA**			
Age (years)	12.3 (10.3, 14.7)	12.2 (10.2, 16.7)	0.571
Hypertension (3 groups)			0.011
Normotensive	25 (63%)	6 (38%)	
High Pressure	2 (5.0%)	6 (38%)	
Hypertensive	13 (33%)	4 (25%)	
Hypertension (2 groups)			0.089
Normotensive	25 (63%)	6 (38%)	
High Pressure/Hypertensive	15 (38%)	10 (63%)	
Systolic Blood Pressure	113 (106, 120)	122 (110, 130)	0.060
Diastolic Blood Pressure	63 (60, 67)	60 (57, 68)	0.760
LVMI (g/m^2^)	44 (38, 52)	65 (42, 72)	0.292
IVSd (cm)	0.71 (0.61, 0.75)	0.75 (0.63, 0.82)	0.416
LVPWd (cm)	0.66 (0.57, 0.77)	0.77 (0.61, 0.85)	0.120
LV 3D GLS (%)	−22.75 (−24.80, −19.00)	−21.20 (−22.60, −20.30)	0.887
LVEF (%)	61.6 (59.6, 64.4)	60.5 (58.6, 65.4)	0.502
TAPSE (cm)	1.50 (1.30, 1.90)	1.60 (1.20, 2.40)	0.687
FAC (%)	45 (41, 49)	45 (39, 50)	0.784
3D LVEF (%)	61.60 (59.80, 62.90)	61.90 (58.80, 65.60)	0.577
3D RVEF (%)	57.7 (50.6, 58.9)	45.9 (43.5, 50.1)	0.076
RVFWLS (%)	−22.4 (−26.3, −21.4)	−21.7 (−23.5, −17.9)	0.374
**COA**			
Age (years)	13.6 (10.4, 16.3)	14.2 (12.1, 16.6)	0.278
Hypertension (3 groups)			0.101
Normotensive	35 (56%)	9 (32%)	
High Pressure	15 (24%)	11 (39%)	
Hypertensive	12 (19%)	8 (29%)	
Hypertension (2 groups)			**0.03**
Normotensive	35 (56%)	9 (32%)	
High Pressure/Hypertensive	27 (44%)	19 (68%)	
Systolic Blood Pressure	112 (101, 125)	120 (111, 126)	0.115
Diastolic Blood Pressure	61 (57, 69)	62 (58, 70)	0.981
LVMI (g/m^2^)	52 (34, 73)	58 (50, 82)	0.264
IVSd (cm)	0.72 (0.61, 0.83)	0.79 (0.65, 0.93)	0.085
LVPWd (cm)	0.68 (0.60, 0.82)	0.73 (0.66, 0.88)	0.080
LV 3D GLS (%)	−22.3 (−25.6, −20.3)	−24.6 (−25.4, −20.0)	0.642
LVEF (%)	63.7 (59.9, 67.2)	60.9 (59.8, 64.5)	0.132
TAPSE (cm)	2.05 (1.65, 2.50)	2.15 (1.70, 2.40)	0.732
FAC (%)	45.9 (42.3, 49.1)	41.7 (40.6, 48.2)	0.279
3D LVEF (%)	64.0 (60.9, 66.6)	60.9 (60.0, 63.7)	0.058
3D RVEF (%)	52.5 (48.6, 56.6)	56.0 (49.3, 61.1)	0.486
RVFWLS (%)	−23.60 (−24.95, −21.60)	−24.50 (−26.40, −23.40)	0.486
**RVOT abnormalities**			
Age (years)	13.7 (10.0, 16.5)	12.3 (9.1, 14.1)	0.1052
Hypertension (3 groups)			**0.042**
Normotensive	61 (69%)	11 (44%)	
High Pressure	19 (22%)	8 (32%)	
Hypertensive	8 (9.1%)	6 (24%)	
Hypertension (2 groups)			**0.020**
Normotensive	61 (69%)	11 (44%)	
High Pressure/Hypertensive	27 (31%)	14 (56%)	
Systolic Blood Pressure	111 (103, 120)	117 (112, 123)	**0.024**
Diastolic Blood Pressure	63 (60, 70)	63 (59, 70)	0.546
LVMI (g/m^2^)	37 (24, 41)	44 (29, 53)	0.110
IVSd (cm)	0.69 (0.60, 0.77)	0.78 (0.63, 0.91)	**0.026**
LVPWd (cm)	0.67 (0.58, 0.77)	0.74 (0.68, 0.83)	**0.053**
LV 3D GLS (%)	−22.8 (−24.5, −20.3)	−21.3 (−23.3, −20.0)	0.449
LVEF (%)	62.0 (60.0, 64.1)	60.2 (59.8, 62.0)	0.149
TAPSE (cm)	1.70 (1.40, 1.90)	1.60 (1.50, 1.80)	0.957
FAC (%)	45.2 (41.1, 48.5)	46.2 (42.5, 48.9)	0.557
3D LVEF (%)	62.4 (60.0, 64.3)	61.1 (59.8, 62.7)	0.225
3D RVEF (%)	52.5 (48.1, 57.1)	49.6 (45.6, 56.1)	0.330
RVFWLS (%)	−23.5 (−26.9, −20.8)	−24.3 (−26.1, −18.8)	0.640
**OTHER**			
Age (years)	13.7 (10.4, 16.3)	13.5 (11.1, 15.8)	0.972
Hypertension (3 groups)			0.342
Normotensive	49 (60%)	17 (47%)	
High Pressure	19 (23%)	9 (25%)	
Hypertensive	14 (17%)	10 (28%)	
Hypertension (2 groups)			0.207
Normotensive	49 (60%)	17 (47%)	
High Pressure/Hypertensive	33 (40%)	19 (53%)	
Systolic Blood Pressure	110 (100, 120)	117 (109, 125)	**0.052**
Diastolic Blood Pressure	64 (60, 67)	65 (60, 70)	0.767
LVMI (g/m^2^)	38 (30, 53)	45 (41, 57)	0.195
IVSd (cm)	0.66 (0.58, 0.72)	0.77 (0.68, 0.85)	**0.003**
LVPWd (cm)	0.62 (0.56, 0.73)	0.71 (0.63, 0.81)	**0.009**
LV 3D GLS (%)	−23.5 (−25.6, −21.2)	−21.7 (−23.2, −20.8)	0.093
LVEF (%)	61.5 (59.1, 65.2)	64.6 (61.4, 66.5)	0.083
TAPSE (cm)	1.80 (1.50, 2.20)	1.90 (1.60, 2.35)	0.633
FAC (%)	41.0 (38.7, 46.6)	45.0 (41.9, 47.0)	0.417
3D LVEF (%)	62.2 (59.9, 64.8)	61.7 (59.4, 63.4)	0.381
3D RVEF (%)	52.7 (44.2, 58.6)	57.8 (57.8, 57.8)	0.750
RVFWLS (%)	−20.5 (−28.9, −20.2)	−24.4 (−31.2, −22.4)	0.267

^1^n (%); Median (Q1, Q3); Categorical variables were compared using Pearson’s chi-squared test, while continuous variables were analyzed using the Kruskal–Wallis test. Legend: LV 3D GLS, left ventricular three-dimensional global longitudinal strain LVEF, left ventricular ejection fraction; 3D LVEF, three-dimensional left ventricular ejection fraction; TAPSE, tricuspid annular plane systolic excursion; FAC, fractional area change; 3D RVEF, three-dimensional right ventricular ejection fraction; RVFWLS, right ventricular free wall longitudinal strain; LVMI, left ventricular mass index; IVSd, interventricular septal thickness in diastole; LVPWd, left ventricular posterior wall thickness in diastole.

**Table 3:** Multivariable association of overweight/obesity with blood pressure, cardiac remodeling, and systolic function in pediatric CHD.

Outcome	Model	N	Effect Estimate for Overweight/Obese vs. Normal Weight	95% CI	*p*-Value	Adjustment
Blood pressure classification						
Blood pressure category, 3 groups	Ordinal logistic regression	377	OR 2.1	1.4 to 3.2	<0.001	CHD diagnosis/severity
Abnormal blood pressure, 2 groups	Binary logistic regression	377	OR 2.3	1.5 to 3.7	<0.001	CHD diagnosis/severity
Hemodynamic parameters						
Systolic blood pressure	Linear regression	377	β 5.1	2.4 to 7.8	<0.001	Age, sex, CHD diagnosis/severity
Diastolic blood pressure	Linear regression	377	β 0.0	−2.0 to 2.1	0.969	Age, sex, CHD diagnosis/severity
Left ventricular structural parameters						
LVMI	Linear regression	322	β 10.0	4.3 to 15.8	<0.001	Age, sex, CHD diagnosis/severity
IVSd	Linear regression	322	β 0.1	0.0 to 0.1	<0.001	Age, sex, CHD diagnosis/severity
LVPWd	Linear regression	322	β 0.1	0.0 to 0.1	<0.001	Age, sex, CHD diagnosis/severity
Ventricular systolic function and deformation						
LV 3D GLS	Linear regression	200	β 0.5	−1.9 to 3.0	0.658	Age, sex, CHD diagnosis/severity
LVEF	Linear regression	308	β −0.4	−1.6 to 0.8	0.555	Age, sex, CHD diagnosis/severity
TAPSE	Linear regression	296	β −0.2	−1.0 to 0.7	0.705	Age, sex, CHD diagnosis/severity
FAC	Linear regression	139	β 0.0	−2.2 to 2.2	0.996	Age, sex, CHD diagnosis/severity
3D LVEF	Linear regression	235	β −1.2	−2.6 to 0.2	0.099	Age, sex, CHD diagnosis/severity
3D RVEF	Linear regression	100	β −2.1	−5.3 to 1.1	0.203	Age, sex, CHD diagnosis/severity
RVFWLS	Linear regression	101	β 0.0	−2.2 to 2.2	0.996	Age, sex, CHD diagnosis/severity

Values are ORs or β coefficients for overweight/obese patients versus normal-weight patients, used as the reference group. Ordinal and binary logistic regression were used for blood pressure categories, and linear regression for continuous outcomes. Linear models were adjusted for age, sex, and CHD diagnosis/severity; logistic models were adjusted for CHD diagnosis/severity only, because pediatric blood pressure categories already account for age, sex, and height. CI, confidence interval; OR, odds ratio. Legend: CHD, congenital heart disease; LVMI, left ventricular mass index; IVSd, interventricular septal thickness in diastole; LVPWd, left ventricular posterior wall thickness in diastole; LV 3D GLS, left ventricular three-dimensional global longitudinal strain; 3D LVEF, three-dimensional left ventricular ejection fraction; TAPSE, tricuspid annular plane systolic excursion; FAC, fractional area change; RVEF, right ventricular ejection fraction; RVFWLS, right ventricular free-wall longitudinal strain; 3D RVEF, three-dimensional right ventricular ejection fraction.

**Table 4:** Multivariable analysis of blood pressure and echocardiographic parameters according to BMI category in pediatric CHD.

Outcome	Model	N	Effect Estimate for Overweight vs. Normal Weight (95% CI), *p*-Value	Effect Estimate for Obese vs. Normal Weight (95% CI), *p*-Value	Adjustment
Blood pressure classification					
Blood pressure category, 3 groups	Ordinal logistic regression	377	OR 1.6 (1.0 to 2.6), *p* = 0.051	OR 3.8 (1.9 to 7.8), *p* < 0.001	CHD diagnosis/severity
Abnormal blood pressure, 2 groups	Binary logistic regression	377	OR 2.0 (1.2 to 3.3), *p* = 0.012	OR 3.6 (1.7 to 8.2), *p* = 0.002	CHD diagnosis/severity
Hemodynamic parameters					
Systolic blood pressure	Linear regression	377	β 3.9 (0.8 to 7.0), *p* = 0.013	β 7.8 (3.5 to 12.1), *p* < 0.001	Age, sex, CHD diagnosis/severity
Diastolic blood pressure	Linear regression	377	β −0.5 (−2.9 to 1.8), *p* = 0.647	β 1.3 (−1.9 to 4.6), *p* = 0.424	Age, sex, CHD diagnosis/severity
Left ventricular structural parameters					
LVMI	Linear regression	322	β 7.1 (2.5 to 11.8), *p* = 0.003	β 15.8 (9.2 to 22.3), *p* < 0.001	Age, sex, CHD diagnosis/severity
IVSd	Linear regression	322	β 0.1 (0.0 to 0.1), *p* < 0.001	β 0.1 (0.0 to 0.1), *p* = 0.003	Age, sex, CHD diagnosis/severity
LVPWd	Linear regression	322	β 0.1 (0.0 to 0.1), *p* < 0.001	β 0.1 (0.0 to 0.1), *p* < 0.001	Age, sex, CHD diagnosis/severity
Ventricular systolic function and deformation					
LV 3D GLS	Linear regression	200	β 0.8 (−1.8 to 3.5), *p* = 0.545	β −0.5 (−5.2 to 4.2), *p* = 0.842	Age, sex, CHD diagnosis/severity
LVEF	Linear regression	308	β −0.3 (−1.7 to 1.0), *p* = 0.644	β −0.5 (−2.5 to 1.5), *p* = 0.647	Age, sex, CHD diagnosis/severity
TAPSE	Linear regression	296	β 0.0 (−1.0 to 1.0), *p* = 0.996	β −0.6 (−2.1 to 0.9), *p* = 0.403	Age, sex, CHD diagnosis/severity
FAC	Linear regression	139	β. −0.4 (−2.8 to 2.0), *p* = 0.722	β 1.8 (−2.7 to 6.3), *p* = 0.424	Age, sex, CHD diagnosis/severity
3D LVEF	Linear regression	235	β −1.6 (−3.2 to 0.0), *p* = 0.053	β −0.2 (−2.6 to 2.3), *p* = 0.901	Age, sex, CHD diagnosis/severity
3D RVEF	Linear regression	100	β −1.6 (−4.9 to 1.7), *p* = 0.341	β −6.8 (−16.3 to2.5), *p* = 0.154	Age, sex, CHD diagnosis/severity
RVFWLS	Linear regression	101	β −0.3 (−2.6 to 2.0), *p* = 0.787	β 3.4 (−3.3 to 10.0), *p* = 0.320	Age, sex, CHD diagnosis/severity

Normal-weight patients were used as the reference group. Effect estimates are presented as odds ratios (ORs) for ordinal and binary logistic regression models and as β coefficients for linear regression models, with 95% confidence intervals (CIs) and *p*-values. Blood pressure category was analyzed using ordinal logistic regression, abnormal blood pressure using binary logistic regression, and continuous hemodynamic and echocardiographic variables using linear regression. Blood pressure models were adjusted for CHD diagnosis/severity, whereas linear regression models were adjusted for age, sex, and CHD diagnosis/severity. BMI, body mass index; CHD, congenital heart disease; LV, left ventricular; LVMI, left ventricular mass index; IVSd, interventricular septal thickness in diastole; LVPWd, left ventricular posterior wall thickness in diastole; LV 3D GLS, left ventricular three-dimensional global longitudinal strain; 3D LVEF, three-dimensional left ventricular ejection fraction; TAPSE, tricuspid annular plane systolic excursion; FAC, fractional area change; 3D RVEF, three-dimensional right ventricular ejection fraction; RVFWLS, right ventricular free-wall longitudinal strain.

## Data Availability

The data presented in this study are available on request from the corresponding author. The data are not publicly available due institutional policies and data protection regulations that do not allow data sharing.

## Abbreviations

The following abbreviations are used in this manuscript:

CHD	Congenital heart disease
ACHD	Adult with congenital heart disease
TGA	Transposition of the great arteries
TOF	Tetralogy of Fallot
PA-IVS	Pulmonary atresia with intact ventricular septum
PVS	Pulmonary valve stenosis
DORV	Double-outlet right ventricle
PA + VDS	Pulmonary atresia with ventricular septal defect
COA	Coarctation of the aorta
TGA + COA	Transposition of the great arteries associated with coarctation of the aorta
BAV	Bicuspid aortic valve
AVS	Aortic valve stenosis
IAA	Interrupted aortic arch
APVR	Anomalous pulmonary venous return
ASD	Atrial septal defect
VSD	Ventricular septal defect
CAV	Complete atrioventricular canal
MVS	Mitral valve stenosis
ccTGA	Congenitally corrected transposition of the great arteries
TAPSE	Tricuspid annular plane systolic excursion
LV 3D GCS	Global circumferential strain
LV 3D GLS	Global longitudinal strain
RVOT	Right ventricular outflow tract

## References

[1] Schwartz S., Olsen M., Woo J.G., Madsen N. (2017). Congenital heart disease and the prevalence of underweight and obesity from age 1 to 15 years: Data on a nationwide sample of children. BMJ Paediatr. Open.

[2] Pinto N.M., Marino B.S., Wernovsky G., de Ferranti S.D., Walsh A.Z., Laronde M., Hyland K., Dunn S.O., Cohen M.S. (2007). Obesity is a common comorbidity in children with congenital and acquired heart disease. Pediatrics.

[3] Steele J.M., Preminger T.J., Erenberg F.G., Wang L., Dell K., Alsaied T., Zahka K.G. (2019). Obesity trends in children, adolescents, and young adults with congenital heart disease. Congenit. Heart Dis..

[4] Perin F., Carreras Blesa C., Rodriguez Vazquez Del Rey M.D.M., Cobo I., Maldonado J. (2019). Overweight and obesity in children treated for congenital heart disease. An. Pediatr..

[5] Avitabile C.M., Leonard M.B., Zemel B.S., Brodsky J.L., Lee D., Dodds K., Hayden-Rush C., Whitehead K.K., Goldmuntz E., Paridon S.M. (2014). Lean mass deficits, vitamin D status and exercise capacity in children and young adults after Fontan palliation. Heart.

[6] Briston D.A., Sabanayagam A., Zaidi A.N. (2017). Observations on obesity patterns in tetralogy of Fallot patients from childhood to adulthood. Cardiol. Young.

[7] Chung S.T., Hong B., Patterson L., Petit C.J., Ham J.N. (2016). High Overweight and Obesity in Fontan Patients: A 20-Year History. Pediatr. Cardiol..

[8] Willinger L., Brudy L., Meyer M., Oberhoffer-Fritz R., Ewert P., Muller J. (2021). Overweight and Obesity in Patients with Congenital Heart Disease: A Systematic Review. Int. J. Environ. Res. Public Health.

[9] Shustak R.J., McGuire S.B., October T.W., Phoon C.K., Chun A.J. (2012). Prevalence of obesity among patients with congenital and acquired heart disease. Pediatr. Cardiol..

[10] Pittrow R.D., Kaemmerer H., Freiberger A., Achenbach S., Bischoff G., Dewald O., Ewert P., Engel A., Freilinger S., Hörer J. (2025). Overweight and Obesity in Adults with Congenital Heart Disease and Heart Failure: Real-World Evidence from the PATHFINDER-CHD Registry. J. Clin. Med..

[11] Chen C.A., Wang J.K., Lue H.C., Hua Y.C., Chang M.H., Wu M.H. (2012). A shift from underweight to overweight and obesity in Asian children and adolescents with congenital heart disease. Paediatr. Perinat. Epidemiol..

[12] Andonian C., Langer F., Beckmann J., Bischoff G., Ewert P., Freilinger S., Kaemmerer H., Oberhoffer R., Pieper L., Neidenbach R.C. (2019). Overweight and obesity: An emerging problem in patients with congenital heart disease. Cardiovasc. Diagn. Ther..

[13] Barbiero S.M., D’Azevedo Sica C., Schuh D.S., Cesa C.C., de Oliveira Petkowicz R., Pellanda L.C. (2014). Overweight and obesity in children with congenital heart disease: Combination of risks for the future?. BMC Pediatr..

[14] Tamayo C., Manlhiot C., Patterson K., Lalani S., McCrindle B.W. (2015). Longitudinal evaluation of the prevalence of overweight/obesity in children with congenital heart disease. Can. J. Cardiol..

[15] Opp D.N., Jain C.C., Egbe A.C., Borlaug B.A., Reddy Y.V., Connolly H.M., Lara-Breitinger K.M., Cordina R., Miranda W.R. (2025). Fontan haemodynamics in adults with obesity compared with overweight and normal body mass index: A retrospective invasive exercise study. Eur. J. Prev. Cardiol..

[16] Fogel M.A., Pawlowski T., Keller M.S., Cohen M.S., Goldmuntz E., Diaz L., Li C., Whitehead K.K., Harris M.A. (2015). The Cardiovascular Effects of Obesity on Ventricular Function and Mass in Patients after Tetralogy of Fallot Repair. J. Pediatr..

[17] Maskatia S.A., Spinner J.A., Nutting A.C., Slesnick T.C., Krishnamurthy R., Morris S.A. (2013). Impact of obesity on ventricular size and function in children, adolescents and adults with Tetralogy of Fallot after initial repair. Am. J. Cardiol..

[18] Aly S., Lizano Santamaria R.W., Devlin P.J., Jegatheeswaran A., Russell J., Seed M., McCrindle B.W. (2020). Negative Impact of Obesity on Ventricular Size and Function and Exercise Performance in Children and Adolescents With Repaired Tetralogy of Fallot. Can. J. Cardiol..

[19] Buelow M.W., Earing M.G., Hill G.D., Cohen S.B., Bartz P.J., Tweddell J.S., Ginde S. (2015). The Impact of Obesity on Postoperative Outcomes in Adults with Congenital Heart Disease Undergoing Pulmonary Valve Replacement. Congenit. Heart Dis..

[20] Radbill A.E., Smith A.H., Van Driest S.L., Fish F.A., Bichell D.P., Mettler B.A., Christian K.G., Edwards T.L., Kannankeril P.J. (2022). Impact of obesity on post-operative arrhythmias after congenital heart surgery in children and young adults. Cardiol. Young.

[21] Vangedal M.S.K., Thuraiaiyah J., Joergensen T.H., Solis A., Langsted A., Nordestgaard B.G., Kistorp C., Raunsoe J., Schmiegelow S.S., Aplin M. (2025). Prevalence of obesity among adult patients with congenital heart disease: A population-based study. Int. J. Cardiol..

[22] Mannarino S., Santacesaria S., Raso I., Garbin M., Pipolo A., Ghiglia S., Tarallo G., De Silvestri A., Vandoni M., Lucini D. (2023). Benefits in Cardiac Function from a Remote Exercise Program in Children with Obesity. Int. J. Environ. Res. Public Health.

[23] Peterson L.R., Soto P.F., Herrero P., Mohammed B.S., Avidan M.S., Schechtman K.B., Dence C., Gropler R.J. (2008). Impact of gender on the myocardial metabolic response to obesity. JACC Cardiovasc. Imaging.

[24] Fabi M., Meli M., Leardini D., Andreozzi L., Maltoni G., Bitelli M., Pierantoni L., Zarbo C., Dondi A., Bertulli C. (2023). Body Mass Index (BMI) Is the Strongest Predictor of Systemic Hypertension and Cardiac Mass in a Cohort of Children. Nutrients.

[25] Brady T.M., Appel L.J., Holmes K.W., Fivush B., Miller E.R. (2016). Association Between Adiposity and Left Ventricular Mass in Children With Hypertension. J. Clin. Hypertens..

[26] Heiskanen J.S., Hernesniemi J.A., Ruohonen S., Hutri-Kähönen N., Kähönen M., Jokinen E., Tossavainen P., Kallio M., Laitinen T., Lehtimäki T. (2021). Influence of early-life body mass index and systolic blood pressure on left ventricle in adulthood—The Cardiovascular Risk in Young Finns Study. Ann. Med..

[27] Simpson S.A., Field S.L., Xu M., Saville B.R., Parra D.A., Soslow J.H. (2018). Effect of Weight Extremes on Ventricular Volumes and Myocardial Strain in Repaired Tetralogy of Fallot as Measured by CMR. Pediatr. Cardiol..

[28] Lewis A.J.M., Abdesselam I., Rayner J.J., Byrne J., Borlaug B.A., Neubauer S., Rider O.J. (2022). Adverse right ventricular remodelling, function, and stress responses in obesity: Insights from cardiovascular magnetic resonance. Eur. Heart J. Cardiovasc. Imaging.

